# Serial Spike Time Correlations Affect Probability Distribution of Joint Spike Events

**DOI:** 10.3389/fncom.2016.00139

**Published:** 2016-12-23

**Authors:** Mina Shahi, Carl van Vreeswijk, Gordon Pipa

**Affiliations:** ^1^Department of Neuroinformatics, Institute of Cognitive Science, University of OsnabrückOsnabrück, Germany; ^2^Centre de Neurophysique, Physiologie et Pathologie, Université René DescartesParis, France

**Keywords:** renewal process, Poisson process, synchrony, ISI, joint spike events, coincidence distribution

## Abstract

Detecting the existence of temporally coordinated spiking activity, and its role in information processing in the cortex, has remained a major challenge for neuroscience research. Different methods and approaches have been suggested to test whether the observed synchronized events are significantly different from those expected by chance. To analyze the simultaneous spike trains for precise spike correlation, these methods typically model the spike trains as a Poisson process implying that the generation of each spike is independent of all the other spikes. However, studies have shown that neural spike trains exhibit dependence among spike sequences, such as the absolute and relative refractory periods which govern the spike probability of the oncoming action potential based on the time of the last spike, or the bursting behavior, which is characterized by short epochs of rapid action potentials, followed by longer episodes of silence. Here we investigate non-renewal processes with the inter-spike interval distribution model that incorporates spike-history dependence of individual neurons. For that, we use the Monte Carlo method to estimate the full shape of the coincidence count distribution and to generate false positives for coincidence detection. The results show that compared to the distributions based on homogeneous Poisson processes, and also non-Poisson processes, the width of the distribution of joint spike events changes. Non-renewal processes can lead to both heavy tailed or narrow coincidence distribution. We conclude that small differences in the exact autostructure of the point process can cause large differences in the width of a coincidence distribution. Therefore, manipulations of the autostructure for the estimation of significance of joint spike events seem to be inadequate.

## 1. Introduction

The mammalian brain is comprised of billions of neurons, each connected to thousands of other neurons by synapses. Every millisecond, thousands of neurons get excited and transmit brief, identical and stereotyped electrical pulses, called action potentials or spikes, to other neurons. The question of what kind of information is embedded in the spike trains, or how sensory and other information is represented in the spike trains and transmitted to other neurons, refers to a well-known puzzle namely, the neural coding problem. This problem has been a longstanding challenge within the neuroscience community and up to the present, there has not been a defined answer to this issue.

To decipher the neural codes, two candidates have largely been examined : 1- rate coding (e.g., Shadlen and Movshon, 1999), 2- temporal coding (e.g., Uhlhaas et al., 2009). Rate coding refers to the scheme which assumes that most, if not all, of the relevant information is transferred via mean firing rate of the neuron that neglects all the information which may exist in the exact timing of spikes. In recent years, the contrary idea of temporal coding has gained increasing attention. The temporal coding hypothesis claims that the temporal patterns of the neural activity may play a role in information coding, i.e., the precise temporal structures of neuronal discharges participate in coding information. Different coding strategies based on spike timing have been proposed, including, the time-to-first-spike (time of the first spike relative to onset of single event), phase-of-firing (phase of the spike with respect to the background oscillation in the brain) and correlations in spike timing of a group of neurons. The first two strategies are mostly concerned with how a single neuron encodes information, and the third strategy, which is known as the correlation coding model, claims that correlations between the spike timing of groups of neurons or cell assemblies convey information.

One extreme case of the correlation is synchrony, by which the spike patterns come in precise millisecond coordination across group of neurons. Coding by synchrony has been studied extensively both experimentally and theoretically (e.g., von der Malsburg, 1981; Gray and Singer, 1989; Gray et al., 1989; Riehle et al., 1997; Vicente et al., 2008; Pipa and Munk, 2011; Haslinger et al., 2013; Toutounji and Pipa, 2014; Torre et al., 2016b). Mounting evidence indicates the importance of neural synchrony in cognitive and executive processes and disruption of synchronization in cognitive dysfunctions (e.g., Niebur et al., 2002; Uhlhaas and Singer, 2006; Haenschel et al., 2007; Palva et al., 2010). Hence, theoretical tools for analyzing synchronized events is a necessity in the field of theoretical neuroscience.

To address the problem of coding by synchrony, one crucial step to take is to determine whether the synchronized events occur above chance. In other words, whether they occur more often than is expected if the individual neurons fire independently. To investigate this issue, different approaches and methods have been taken (e.g., Aertsen et al., 1989; König, 1994; Grün et al., 1999; Grün et al., 2002a,b; Pipa and Grün, 2003; Pipa et al., 2007, 2008; Staude et al., 2010; Torre et al., 2013, 2016a). To analyze ensembles of spike trains from simultaneously recorded neurons for precise spike correlations, many of these approaches model the spike train as a Poisson process with the same rate profile as the neuron under investigation (e.g., Grün et al., 2002a,b). Poisson process is a memoryless process, i.e., the occurrence of a spike at time *t* does not depend on the time occurrence of the previous spikes. In other words, the generation of each spike is independent of all other spikes. Another characteristic of the spike trains modeled as a Poisson process is that the inter-spike interval (ISI) follows an exponential distribution. However, the experimental spike trains show substantial deviation from these characteristics of Poisson process, i.e., independence of spike times and an exponential ISI distribution (e.g., Burns and Webb, 1976; Levine, 1991; Iyengar and Liao, 1997; Teich et al., 1997; Krahe and Gabbiani, 2004; Nawrot et al., 2007, 2008; Farkhooi et al., 2009). For example, neural spike trains exhibit absolute and relative refractory periods during which the probability of oncoming action potential, based on the time of the last spike, is zero or very low, respectively, or the bursting behavior, which is characterized by short epochs of rapid action potentials, followed by longer episodes of silence.

To model such characteristics of the neural firing, i.e., refractoriness, burstiness, and the regularity of the spike trains, Pipa et al. (2013) used two types of renewal processes, namely, a gamma process and a log-normal process, to study the effects of the autostructure of the spike trains on the shape of the probability distribution of coincidence count distribution of pairs of mutually independent spike trains. It is shown that the width of the coincidence count distribution depends on detailed properties of the ISI distribution, such as the coefficient of variation *C*_*V*_.

Assuming mutually independent ISIs, Pipa et al. (2013) used a renewal process to model the autostructure of the spike trains. However, this assumption may not be always in agreement with the characteristics of the biological spike trains. Neural firing might be described by non-renewal processes which can model higher order dependence of spike times that lie further back in the past (Nawrot et al., 2007). We will show that the higher order dependence can be modeled by a non-renewal process. We will further investigate the impact of the higher order dependence of the spike times on the probability distribution of coincidence events.

## 2. Materials and methods

### 2.1. Renewal process

Renewal process is a simple class of point process model with a very rich mathematical structure that can be an appropriate candidate to model events that occur randomly in time or space.

**Definition 1**. Renewal process *is a stochastic process to models the random events in time (space) that are independent and identically distributed. It is called renewal because the process starts over after each event occurs, and the only factor that affects the likelihood of occurrence of an event is the elapsed time (space) since the last event*.

Renewal process is frequently used to model the spike train, which implies that the inter-spike interval is i.i.d and the probability of occurrence of a spike depends on the elapsed time since the last spike. However, the occurrences of other spikes in the past do not affect the generation of the oncoming spike, in other words, if λ is an instantaneous firing rate at time *t* and *H*(*t*) is the history of the spikes and *t*_*N*(*t*)_ is the time of the last spike then:

(1) $$\begin{array}{l} \lambda \left(t|H\left(t\right)\right)=\lambda \left(t-t_{N\left(t\right)}\right) \end{array}$$

#### 2.1.1. Poisson process

Poisson process is a simple renewal process where the time between the successive arrivals is distributed exponentially, i.e.,

(2) $$\begin{array}{l} P\left\{X=x\right\}=\lambda e^{-\lambda x}; \end{array}$$

where *x*, λ ≥ 0. Since Poisson process is a memoryless process, the probability of occurrence of a new spike does not depend on the elapsed time since the last spike. For a homogenous Poisson process, the instantaneous firing rate is given by:

(3) $$\begin{array}{l} \lambda \left(t|H\left(t\right)\right)=\lambda \end{array}$$

and for an inhomogenous Poisson process, the instantaneous firing rate is as follows:

(4) $$\begin{array}{l} \lambda \left(t|H\left(t\right)\right)=\lambda \left(t\right) \end{array}$$

Two other renewal processes widely used in modeling the spike trains are gamma and log-normal processes with the inter-spike intervals distributed from gamma and log-normal probability distribution, respectively.

#### 2.1.2. Gamma process

The gamma probability distribution is a two parameter probability distribution defined as follows:

(5) $$\begin{array}{r} p\left(x;\beta ,\theta \right)=\frac{\beta^{\theta }e^{-\beta x}}{\Gamma \left(\theta \right)}x^{\theta -1} \end{array}$$

(6) $$\begin{array}{r} E\left[X\right]=\int_{0}^{+\infty }xp\left(x\right)dx=\frac{\theta }{\beta } \end{array}$$

(7) $$\begin{array}{r} \mathrm{Var}\left[X\right]=E\left[X^{2}\right]-{\left(E\left[X\right]\right)}^{2}=\frac{\theta }{\beta^{2}} \end{array}$$

θ and β are two parameters that describe the shape and the rate of the gamma process, respectively.

Given a constant firing rate λ = *R* and an average inter-spike interval $\langle X\rangle =\frac{1}{R}$, two properties of the gamma process with the parameters θ and β are as follows:

(8) $$\begin{array}{r} \beta =\theta R \end{array}$$

(9) $$\begin{array}{r} C_{V}^{2}=\frac{\sigma_{X}^{2}}{\langle X\rangle^{2}}=\frac{1}{\theta } \end{array}$$

since $\sigma_{X}^{2}=\frac{1}{\theta R^{2}}$ and $\langle X\rangle =\frac{1}{R}$. *C*_*V*_ is the coefficient of variation of the inter-spike interval distribution and it is one of the factors that is widely used to characterize the autostructure of the spike trains. Thus, gamma spike trains can be either described by the shape parameter θ and the rate β or with the coefficient of variation of the inter-spike interval *C*_*V*_ and the firing rate *R*.

Poisson process is a special case of the gamma process for which θ = 1 and ISI distribution is exponential. The distribution of ISI becomes hyperexponential for a shape parameter θ < 1, which makes the short intervals more likely to happen than for a Poisson process with the same firing rate. Gamma process with θ < 1 can be used to model the bursty spike trains which is characterized by short epochs of rapid action potentials, followed by longer episodes of silence. On the contrary, when θ > 1, the gamma distribution approaches a narrow normal distribution which can be used to model regular spike trains (Pipa et al., 2013).

#### 2.1.3. Log-normal process

If {*X*} is a random variable which is log-normally distributed with two parameters *a* and *k* then:

(10) $$\begin{array}{r} \ln\;N\left(x;a,k\right)=\frac{1}{k\sqrt{2\pi }}\frac{\text{exp}\left(-\frac{{\left(\ln\left(x\right)-a\right)}^{2}}{2k^{2}}\right)}{x} \end{array}$$

(11) $$\begin{array}{r} E\left[X\right]=\int_{0}^{+\infty }x\ln\;N\left(x\right)dx=e^{a+\frac{k^{2}}{2}} \end{array}$$

(12) $$\begin{array}{r} \mathrm{Var}\left[X\right]=E\left[X^{2}\right]-{\left(E\left[X\right]\right)}^{2}=\left(e^{k^{2}}-1\right)e^{2a+k^{2}} \end{array}$$

Given a constant firing rate *R* and a coefficient of variation of inter-spike interval *C*_*V*_ two parameters *a* and *k* can be expressed as follows:

(13) $$\begin{array}{r} a=-\ln\;R-\frac{1}{2}\ln\left(C_{V}^{2}+1\right) \end{array}$$

(14) $$\begin{array}{r} k=\sqrt{\ln\left(C_{V}^{2}+1\right)} \end{array}$$

The log-normal distribution of the inter-spike intervals is more heavy-tailed than the inter-spike intervals distributed according to the gamma distribution. Also, very short intervals are unlikely to happen, which makes this distribution a good candidate to model the refractory period when short inter-spike intervals on the order of several milliseconds are unlikely to happen.

### 2.2. Non-renewal process

One further step to generalize a stochastic process model to generate the spike trains is to remove the property of independence of the inter-spike intervals, as abundant experimental observations have shown that the spiking activity of many neurons cannot be modeled as a renewal process, since the occurrence of an action potential depends on other action potentials that occurred in the past (Burns and Webb, 1976; Levine, 1991; Iyengar and Liao, 1997; Teich et al., 1997; Krahe and Gabbiani, 2004; Nawrot et al., 2007, 2008; Farkhooi et al., 2009). In other words, they display history dependence in their spiking activity that persists over multiple action potential firings (Perkel et al., 2011). To model such spiking activities, a non-renewal process (denoted as C-log-normal process) is introduced in this paper. To this end, a new ISI probability distribution, called the C-log-normal probability distribution, which is a generalized form of the log-normal probability distribution, is presented. In the following section, a formal definition of the C-log-normal probability distribution and its properties are given.

#### 2.2.1. C-log-normal process

**Definition 2**. C-log-normal probability distribution is a doubly-stochastic Gaussian process with log-normal intensity, and it is obtained as follows:

1. Let *X*_*n*_ ~ *N*(0, 1), *X*_*n*−1_ ~ *N*(0, 1) and $\zeta_{n}\sim N\left(0,1-\gamma^{2}\right),|\gamma |<1,\gamma \neq 0$
2. Define *X*_*n*_ = γ*X*_*n*−1_ + ζ_*n*_
3. Define $Z_{n}=\frac{X_{n}-\alpha X_{n-1}}{\sqrt{1+\alpha^{2}-2\alpha \gamma }},\left(\alpha \left(\alpha -2\gamma \right)>-1\right)$
4. Define $t_{n}=e^{a+kZ_{n}}.$

The marginal distribution of C-log-normal probability distribution is defined as follows:

(15) $$\begin{array}{l} \ln\;N{\left(t_{n};a,k,\alpha ,\gamma \right)}_{C-log-normal}=\frac{1}{k\sqrt{2\pi }}\frac{exp\left(-\frac{{\left(ln\left(t_{n}\right)-a\right)}^{2}}{2k^{2}}\right)}{t_{n}}, \end{array}$$

where *a*, *k*, α, and γ are the parameters of the distribution. The difference between the log-normal process and the C-log-normal process is given in the following example.

**Example:** Let *X*_*n*_ be an independent random variable with a normal distribution, i.e., $X_{n}\sim N\left(0,1\right)$. Then *T*_*n*_ = *exp*(*X*_*n*_) follows a log-normal distribution. However, *X*_*n*_s from the C-log-normal probability distribution are not independent variables. If we define *X*_*n*_ and *Z*_*n*_ according to definition 2 (2) and 2 (3) respectively, then *T*_*n*_ = *exp*(*Z*_*n*_) has a C-log-normal distribution. Bare in mind that here, $Z_{n}\sim N\left(0,1\right)$ (See Supplement 2.1 and 2.2). However, they are not independent.

**Figure 1:**
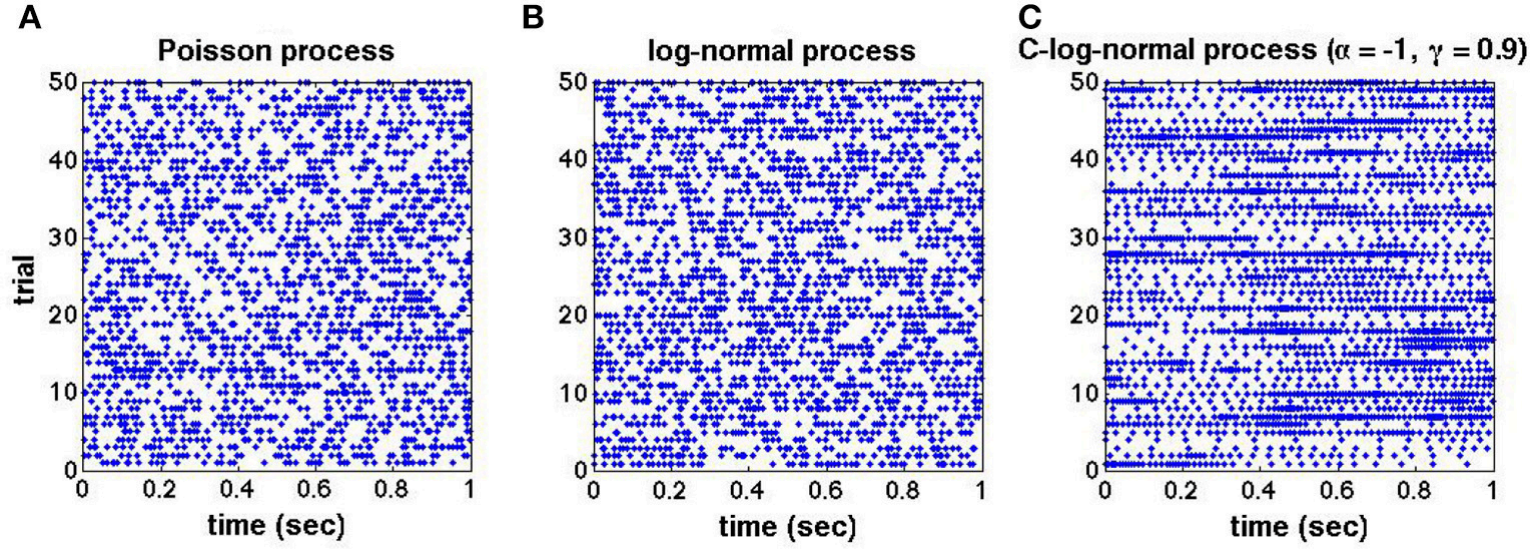
**(A–C)** Raster plots for 50 trials of mutually independent spike trains generated from the Poisson, log-normal, and C-log-normal processes, respectively. Spike rate for all spike trains are chosen to be *R* = 50*Hz* and *CV* = 1.

To illustrate the effect of dependent ISIs on the spike train's autostructure, we compare the spike trains generated from the Poisson, log-normal and C-log-normal processes. Figures 1A–C shows the raster plots for 50 trials of mutually independent spike trains from the aforementioned processes, respectively. The spike rate for all three processes is 50*Hz*, however, the autostructure of the spike trains is different in the C-log-normal process. That is, the burst firings with longer periods happen more often in the C-log-normal process than in the other two processes. The next section provides a more in-depth look at the properties of C-log-normal processes.

#### 2.2.2. Properties of the C-log-normal process

**Figure 2:**
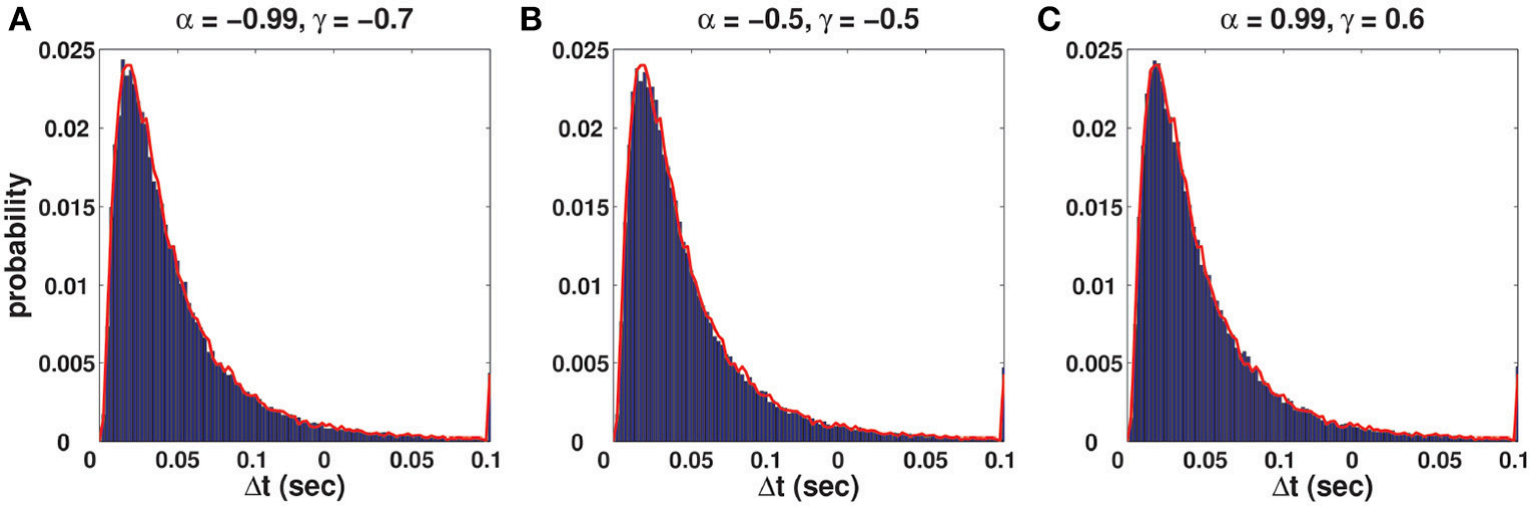
**(A–C)** Solid distribution shows the ISI distribution of the C-log-normal process for different pairs of α and γ. The red curve illustrates the ISI distribution of the log-normal process. For all distributions *CV* = 1, *R* = 50*Hz*.

**Figure 3:**
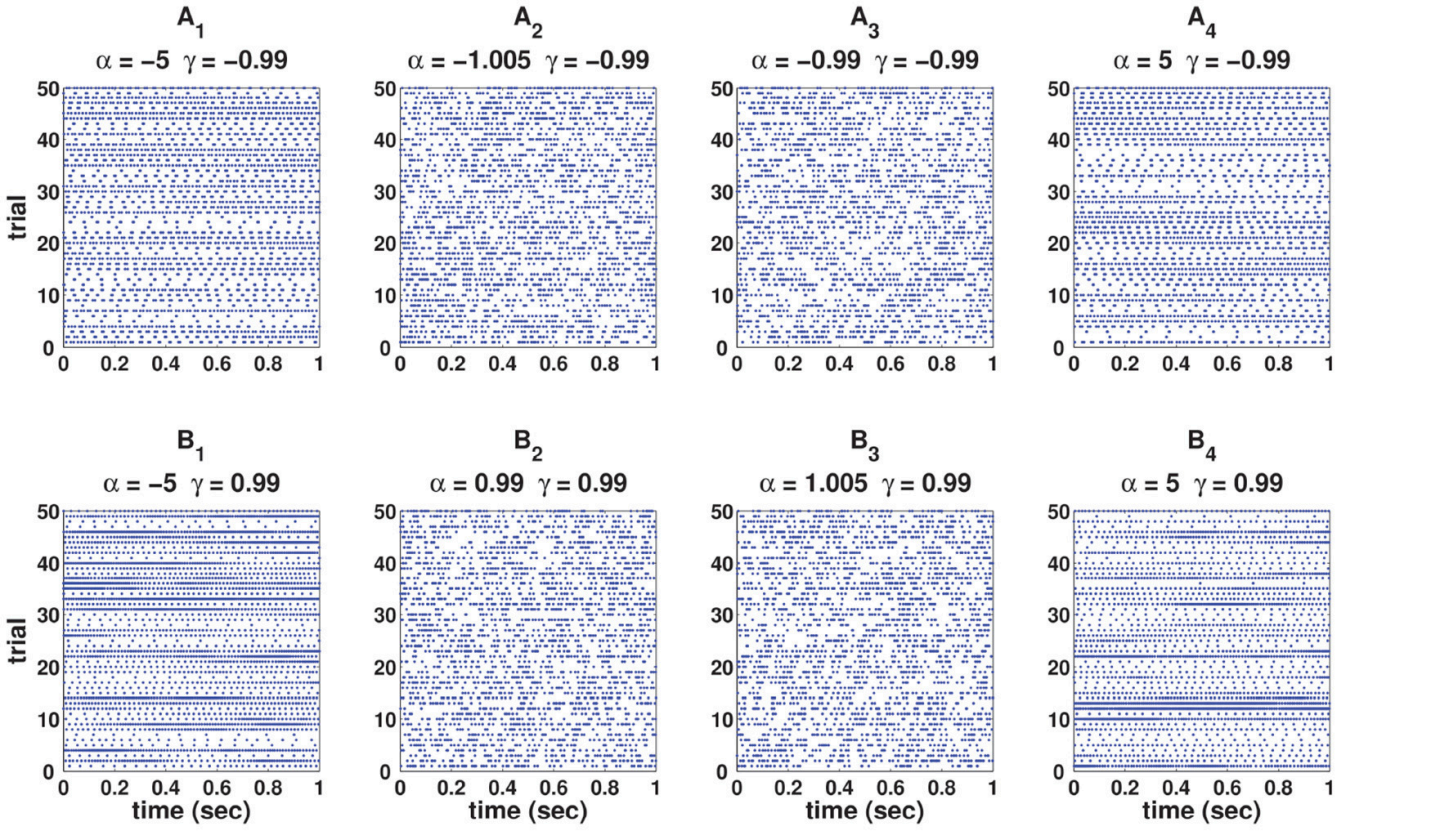
**Eight spike rasters, each has 50 mutually independent spike trains generated from C-log-normal process for different pairs of γ and α**. For all distributions *CV* = 1, *R* = 50*Hz*. **(A**_1_**–A**_4_**)** correspond to γ < 0 and **(B**_1_**–B**_4_**)** correspond to γ > 0.

**Figure 4:**
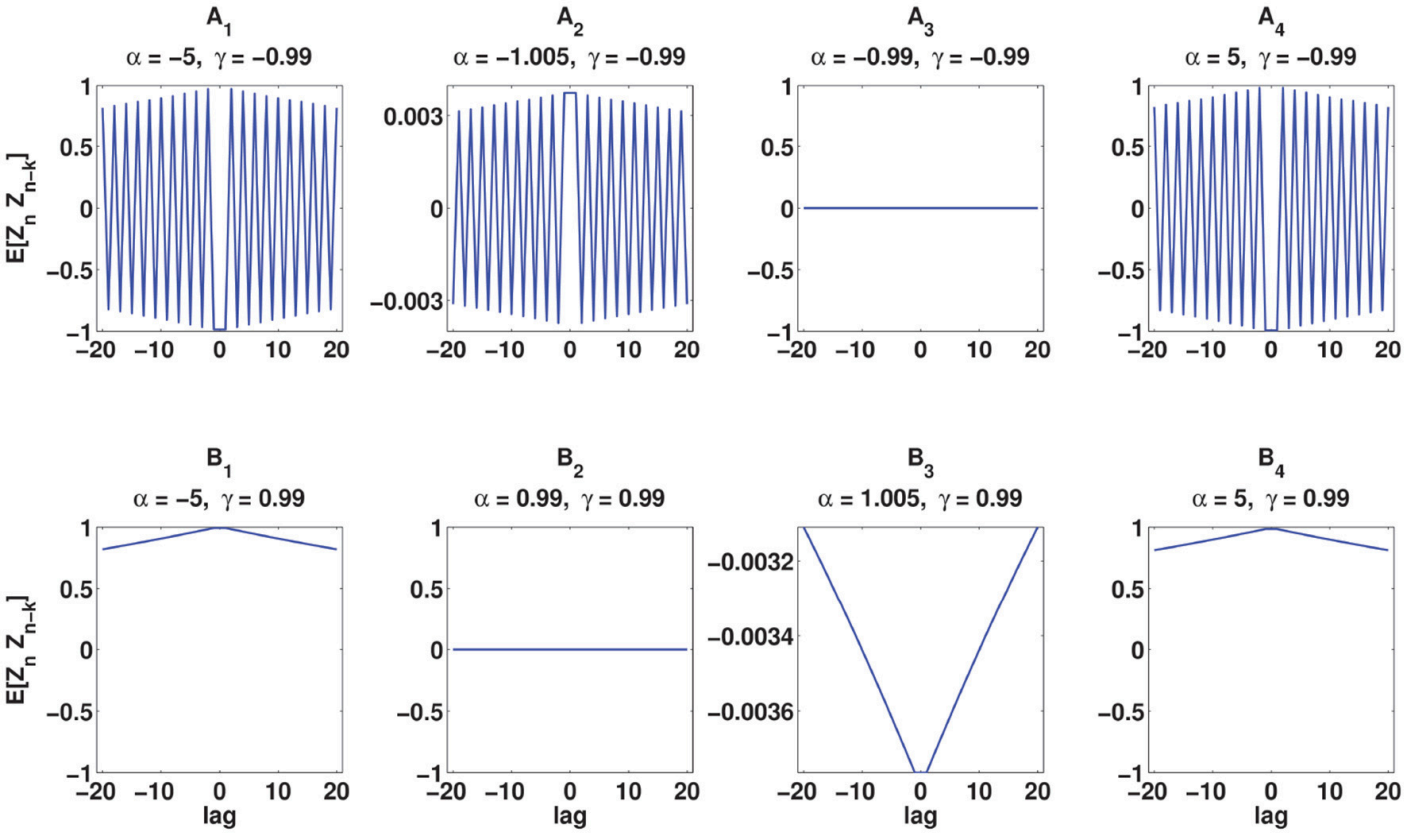
**Analytical E[*Z*_*n*_*Z*_*n*−*k*_] (Equation 26) for the same pairs of γ and α used in Figure 3**. **(A**_1_**–A**_4_**)** correspond to γ < 0 and **(B**_1_**–B**_4_**)** correspond to γ > 0.

- If $X_{1}\sim N\left(0,1\right)$, then the successive *X*_*i*_s obtained by definition 2 (2) are also normally distributed, i.e., $X_{i}\sim N\left(0,1\right)$ (See Supplement 2.1 for the proof).
- *Z*_*n*_ given by definition 2 (3) has a normal distribution, i.e., $Z_{i}\sim N\left(0,1\right)$ (See Supplement 2.2 for the proof).
- If *Z*_*n*_ and *Z*_*n*−*k*_ are defined by definition 2 (3), then
  (16) $$\begin{array}{r} <Z_{n}Z_{n-k}>=\gamma^{|k|-1}\left(\frac{\left(1+\alpha^{2}\right)\gamma -\alpha \left(1+\gamma^{2}\right)}{1+\alpha^{2}-2\alpha \gamma }\right),\\ k\neq 0;\left(\alpha \left(\alpha -2\gamma \right)>-1\right) \end{array}$$
  (See Supplement 2.3 for the proof).
- The log normal process is a special case of the C-log-normal process, when α = γ, which in this case:
  $$<Z_{n}Z_{n-k}>=0.$$
  That is, there is no dependence between *Z*_*n*_s, which results in mutually independent ISIs of a spike train, and that is the property of the log-normal process.
- C-log-normal and log-normal processes have the same ISI distribution. However, the ISIs of the C-log-normal process are correlated but the ISIs of the log-normal process are independent. Figure 2 illustrates the ISI distribution of the C-log-normal process for different pairs of α and γ (parameters of C-log-normal process). The solid distribution is the ISI distribution of the C-log-normal process, while the red curve indicates the ISI distribution of the log-normal process. Both ISI distributions have the same profile.
- Since the ISI distribution of C-log-normal and log-normal process are the same, the first order statistics of their ISIs, such as the mean and variance of their ISIs, are also equal. Thus, the parameters *a* and *k* of C-log-normal process can be substituted by the spike rate *R* and the coefficient of variance *C*_*V*_ according to Equation (2.4.2.4) and (2.4.2.5), respectively.
- The values of α and γ affect the autostructure of the spike train. Figure 3 shows 8 spike rasters, each has 50 mutually independent spike trains generated from the C-log-normal process for different pairs of γ and α. For the same pairs of γ and α, Figure 4 illustrates E[*Z*_*n*_*Z*_*n*−*k*_] defined in Equation (26). For a negative γ, E[*Z*_*n*_*Z*_*n*−*k*_] oscillates between negative and positive values. If α_1_ < α < α_2_^1^ then oscillation starts from a positive value (first lag) (Figure 4A_2_). If α < α_1_ or α > α_2_ then oscillation is in the opposite order, it starts from a negative and then to a positive (Figures 4A_1_,A_4_). For a positive γ, E[*Z*_*n*_*Z*_*n*−*k*_] does not oscillate and it is either positive or negative depending on the value of α. If α_1_ < α < α_2_, then E[*Z*_*n*_*Z*_*n*−*k*_] is negative (Figure 4B_3_), and if α < α_1_ or α > α_2_ it is positive for all lags (Figures 4B_1_,B_4_). Table 1 summarizes the results. Refer to the Figure S1 for the autocorrelation of the spike times.

**Table 1:** **Effects of parameters α and γ on the sign of E[*Z*_*n*_*Z*_*n*−*k*_]**.

	**α_1_ < α < α_2_**	**α > α_1_ or α < α_2_**
γ < 0	E[*Z*_*n*_*Z*_*n*−*k*_] oscillates from positive to negative	E[*Z*_*n*_*Z*_*n*−*k*_] oscillates from negative to positive
γ > 0	E[*Z*_*n*_*Z*_*n*−*k*_] < 0	E[*Z*_*n*_*Z*_*n*−*k*_] > 0

### 2.3. Simulation methods

#### 2.3.1. Coincidence count distribution

To estimate the coincidence count distribution, the following procedure is applied:

1. Generate *N* = 600 mutually independent spike trains of length *T* = 5 s from the C-log-normal distribution.
2. Divide the length of the first spike train into *N*_*bin*_ exclusive bins with the length of Δ*t* = 4 ms, thus the total number of bins, *N*_*bin*_, is $\frac{T}{\Delta t}.$
3. For each spike train, count the number of spikes that fall into each bin. $n_{i}^{j}\left(i=1,2,\ldots ,\frac{T}{\Delta t}\right)$, is the number of the spikes that are assigned to the bin *i* from the *j*^*th*^ spike train.
4. Count the number of coincidences for each independent pair of spike trains *a* and *b*, that is, multiply the number of the spikes in the bin *i* of the spike train *a*, i.e., $n_{i}^{a}$, by the number of the spikes in the bin *i* of te spike train *b*, i.e., $n_{i}^{b}$. Repeat this step for all the bins. The number of coincidences of these two spike trains is the sum of the number of coincidences of all the bins, i.e., $N_{c}=\overset{N_{bin}}{\underset{i=1}{\sum }}n_{i}^{a}n_{i}^{b}.$
5. Generate a histogram of *N*_*c*_s as an estimate of the coincidence count distribution.

#### 2.3.2. Estimation of false positive rate

To estimate the false positive rate of the coincidence count distribution of C-log-normal process, we take the following steps:

1. Generate *N*_1_ = 200 mutually independent spike trains of length *T* = 5 s and firing rate of *R* = 50 Hz from the Poisson process.
2. Compute the coincidence count distribution, *f*_*p*_, according to the Section Coincidence Count Distribution.
3. Estimate the expected number the coincidence counts, λ, by taking the mean *f*_*p*_.
4. Find the empirical estimation of *f*_*p*_, *F*_*p*_.
5. Generate *N*_2_ = 1000 random numbers from the Poisson probability distribution with rate λ.
6. Estimate the empirical cumulative distribution function, *F*_*p*_, of *f*_*p*_.
7. Use *F*_*p*_ and find the critical number, *N*_*criticalPoisson*_, for which 1 − *F*(*N*_*criticalPoisson*_) < 1%. This critical number corresponds to 1% significance level.
8. Set *i* = 1.
9. Generate *N* = 200 mutually independent spike trains of length *T* = 5 s and firing rate of *R* = 50 Hz from C-log-normal process.
10. Compute the coincidence count distribution, *f*_*p*_, according to the Section Coincidence Count Distribution.
11. Find the empirical estimation of *f*_*C*−*logN*_, *F*_*C*−*logN*_.
12. Use *F*_*C*−*logN*_ and *N*_*criticalPoisson*_ to compute:
  (17) $$\begin{array}{l} FP=1-F_{C-logN}\left(N_{criticalPoisson}\right). \end{array}$$
13. *FP* is the false positive rate of coincidence count distribution of C-log-normal process corresponds to 1% significance level under the assumption that the underlying spike trains are generated from Poisson process.
14. Iterate steps 9–12 for *i* = 2, …, 10 trials.
15. Iterate steps 1–14 and substitute the Poisson process with the Log-Normal process.

## 3. Results

This section presents the effects of parameters of the C-log-normal process, namely, α and γ, on the autostructure of the spike trains. The questions addressed in this section are: 1-how do parameters α and γ influence the autocorrelation of C-log-normal process 2- what is the role of parameters α and γ on the order of serial correlation of ISIs? 3- how so parameters α and γ affect the shape of the coincidence count distribution? and finally, how do values of α and γ influence the false positive rate for a particular test level?

### 3.1. Effect of α and γ on the ISI correlation

**Figure 5:**
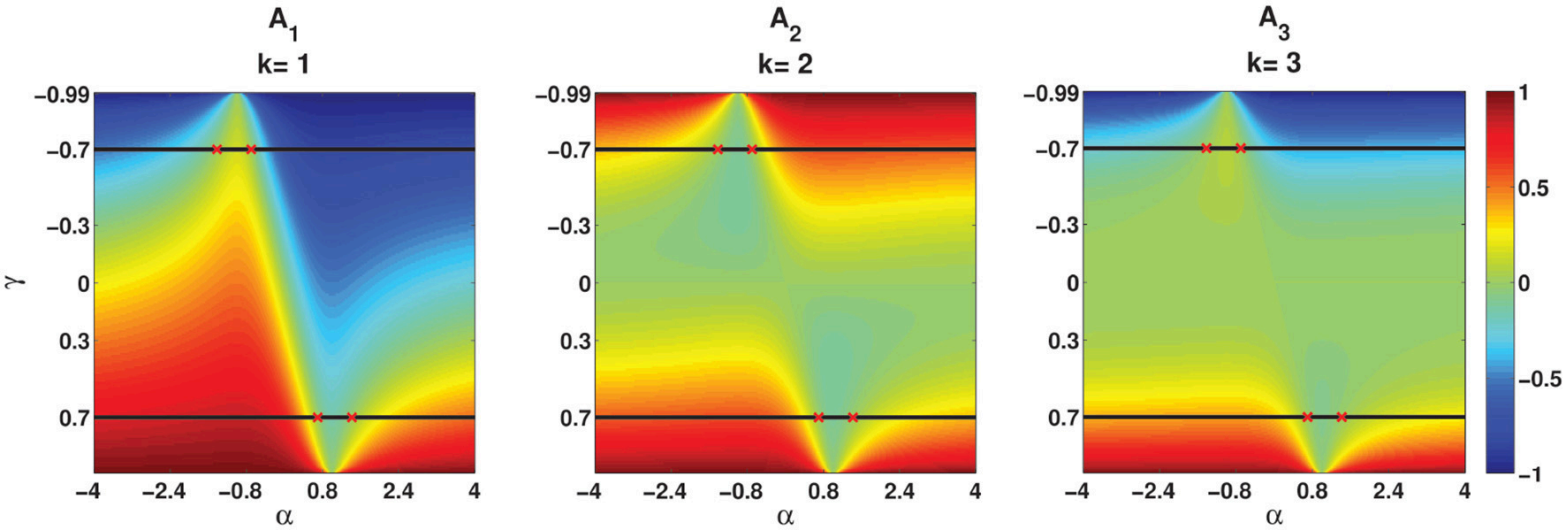
**Analytical E[*Z*_*n*_*Z*_*n*−*k*_] (Equation 26) for different values of parameters (α, γ). (A_1_–A_3_)** Correspond to *k* = 1, *k* = 2, and *k* = 3, respectively. Red crosses indicate the values of α which are the solutions of E[*Z*_*n*_*Z*_*n*−*k*_] = 0 for γ = −0.7 and γ = −0.7.

Figure 5 illustrates in the form of a colormap of a comparison E[*Z*_*n*_*Z*_*n*−*k*_] for different values of parameters α and γ. Figures 5A_1_– A_3_ show E[*Z*_*n*_*Z*_*n*−*k*_] for *k* = 1, *k* = 2, and *k* = 3, respectively. Two examples, namely γ = −0.7 and γ = 0.7 which are indicated by two black lines, are considered to be explained in more detail in this part. Two solutions of E[*Z*_*n*_*Z*_*n*−*k*_] = 0 when γ = −0.7 and γ = 0.7 are [α_1_ = −1.42, α_2_ = −0.7] and [α_1_ = 0.7, α_2_ = 1.42], respectively. α_1_s and α_2_s are shown by red crosses in Figures 5A_1_–A_3_. First consider the value of E[*Z*_*n*_*Z*_*n*−*k*_] for γ = −0.7. As it is summarized in Table 1, for γ < 0 and α_1_ < α < α_2_, E[*Z*_*n*_*Z*_*n*−*k*_] oscillates from a positive to a negative value. The same results are shown in Figures 5A_1_–A_3_. For α_1_ < α < α_2_, E[*Z*_*n*_*Z*_*n*−*k*_] is positive for *k* = 1, negative for *k* = 2, and it is positive for *k* = 3. For α < α_1_ or α > α_2_ the oscillation is in the opposite order, i.e., E[*Z*_*n*_*Z*_*n*−*k*_] is negative for *k* = 1, positive for *k* = 2, and for *k* = 3 is negative.

In the second case when γ = 0.7, E[*Z*_*n*_*Z*_*n*−*k*_] does not oscillate and it is either positive or negative based on the value of α. As given in Table 1, for γ > 0 and α_1_ < α < α_2_, E[*Z*_*n*_*Z*_*n*−*k*_] is negative and for α < α_1_ or α > α_2_ it is positive, irrespective of the value of *k*. As indicated in Figures 5A_1_–A_3_ for γ = 0.7, E[*Z*_*n*_*Z*_*n*−*k*_] is negative where α_1_ < α < α_2_ and it is positive where α < α_1_ or α > α_2_.

Moreover, the effects of the values of α and magnitude of γ are illustrated in Figure 5. The more α is away from one side of the interval, the higher the absolute value of E[*Z*_*n*_*Z*_*n*−*k*_], and the bigger the magnitude of γ is, the more *Z*_*n*_s are correlated to the previous *Z*_*n*−*i*_s and the correlation goes further into the past.

Another characteristic that can be observed by comparison of the magnitude of E[*Z*_*n*_*Z*_*n*−*k*_] in Figures 5A_1_–A_3_ is that the magnitude of E[*Z*_*n*_*Z*_*n*−*k*_] is higher for *k* = 1 for different parameters of αs and γs. Whereas, E[*Z*_*n*_*Z*_*n*−*k*_] in Figures 5A_2_,A_3_ is mostly indicated by green, which, has the range of [−0.1–0.2]. That is, the bigger *k* is, the smaller the magnitude of E[*Z*_*n*_*Z*_*n*−*k*_].

The previous section presented the relation between parameters α and γ and E[*Z*_*n*_*Z*_*n*−*k*_]. However, the roles of α and γ in ISI serial correlation has not yet been and will be covered in this section.

**Figure 6:**
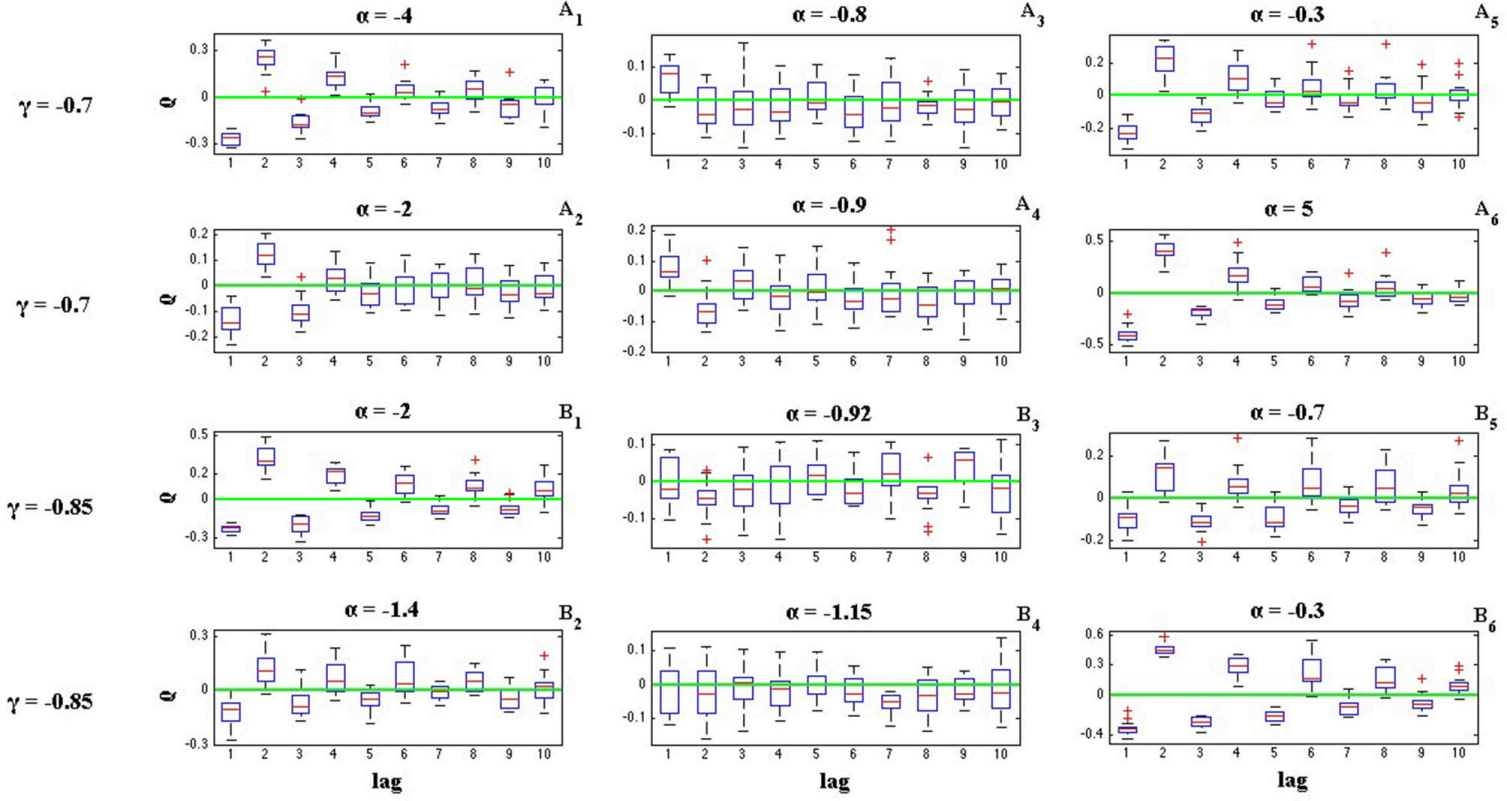
**Effects of parameters α and γ on the ISI serial correlation of the C-log-normal process**. The first column corresponds to α < α_1_, the second column corresponds to α_1_ < α < α_2_, and the third column corresponds to α > α_2_, where α_1_ and α_2_ are the solutions for E[*Z*_*n*_*Z*_*n*−*k*_] = 0 for a given parameter γ (*CV* = 1, *R* = 50*Hz*). **(A**_1_**–A**_6_**)** correspond to γ = −0.7 and different α. **(B**_1_**–B**_6_**)** correspond to γ = −0.85 and different α.

**Figure 7:**
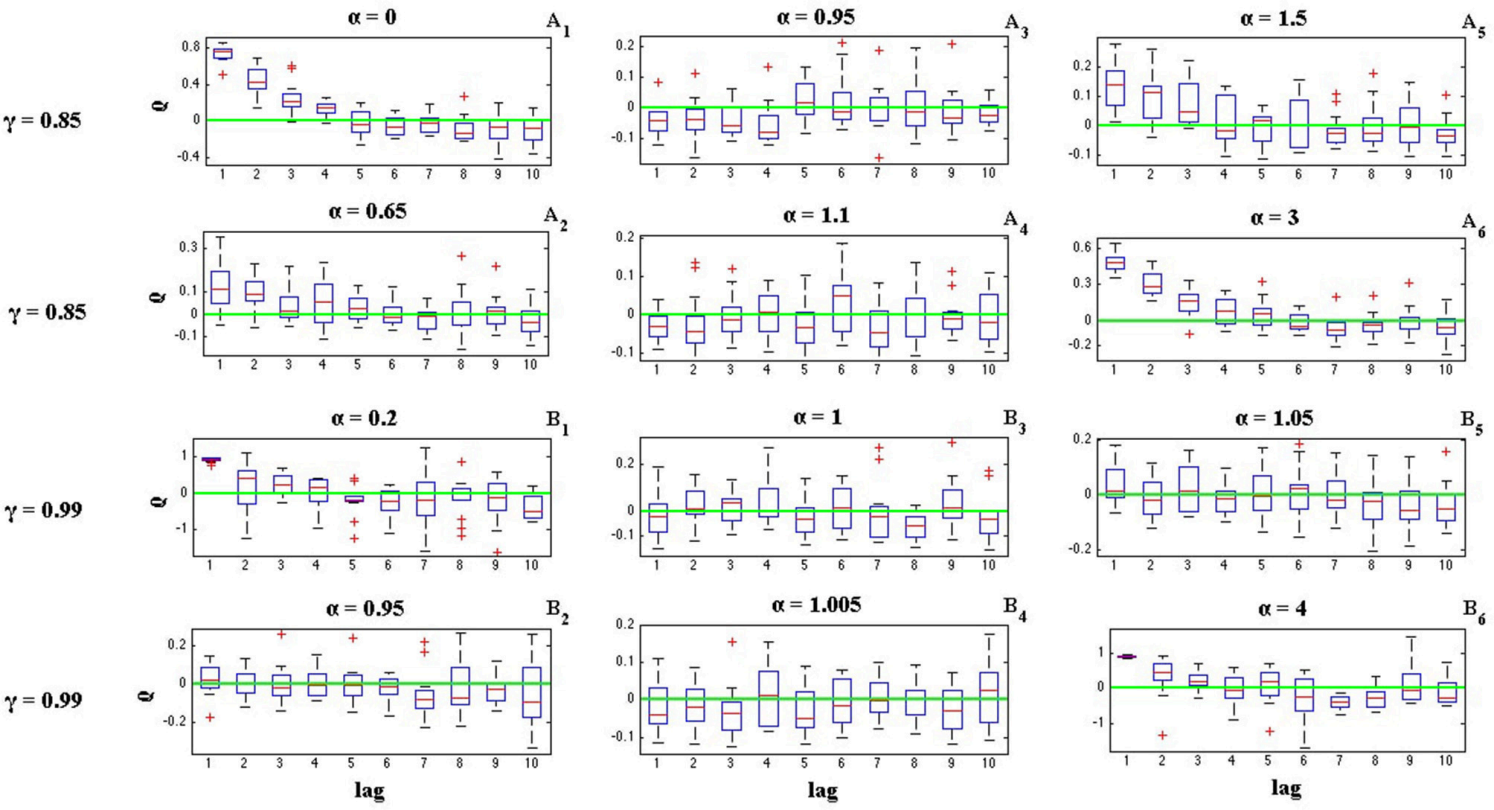
**Effects of parameters α and γ on the ISI serial correlation of the C-log-normal process**. The first column corresponds to α < α_1_, the second column corresponds to α_1_ < α < α_2_, and the third column corresponds to α > α_2_, where α_1_ and α_2_ are the solutions for E[*Z*_*n*_*Z*_*n*−*k*_] = 0 for a given parameter γ (*CV* = 1, *R* = 50*Hz*). **(A**_1_**–A**_6_**)** correspond to γ = 0.85 and different α. **(B**_1_**–B**_6_**)** correspond to γ = 0.99 and different α.

The correlation between ISI_*i*_ and ISI_*i*+*j*_ is quantified by the ISI serial correlation coefficient, ρ_*j*_, where *j* is the lag. ρ_*j*_ is given as follows:

(18) $$\begin{array}{l} \rho_{j}=\frac{\langle I_{i}I_{i+j}\rangle -\langle I_{i}\rangle^{2}}{\langle I_{i}^{2}\rangle -\langle I_{i}\rangle^{2}}, \end{array}$$

where *I*_*i*_ is the length of *i*^*th*^ inter-spike interval. Figures 6, 7 illustrate the effects of α and γ on the ISI serial correlation. Each box plot corresponds to *N* = 15 spike trains of length *T* = 5 s. The green line indicates ρ_*j*_ = 0 as the base line.

The effects of α and γ on the ISI correlation coefficient, are in the same way as they are on E[*Z*_*n*_*Z*_*n*−*k*_]. For negative γs, ρ_*j*_ oscillates between positive and negative values and if α < α_1_ or α > α_2_, then the ISI correlation coefficient for *j* = 1 is negative and for *j* = 2 is positive and so on. However, if α_1_ < α < α_2_ then the sequence of ρ_*j*_ oscillates in the opposite order, it is first positive and then negative and so on. For positive γ, ρ_*j*_ does not show any oscillatory behavior and it is either positive or negative depends on the value of α (Figure 7). It is positive if α < α_1_ or α > α_2_ and it is negative if α_1_ < α < α_2_.

Figures 6, 7 also show the effect of the value of α on the ISI serial correlation. The more α is away from one side of the interval [α_1_, α_2_] the higher the value of ρ_*j*_. For example, Figures 7A_1_,A_2_ compare the values of ρ_*j*_ for parameters γ = 0.85, α = 0 and γ = 0.85, α = 0.65, respectively. Since for γ = 0.85 two solutions of E[*Z*_*n*_*Z*_*n*−*k*_] = 0 are α_1_ = 0.85 and α_2_ = 1.176, and |0−0.85| > |0.65−0.85|, ρ_*j*_ shows higher value for α = 0. For each value of γ shown in Figures 6, 7, first column corresponds to α < α_1_, second column corresponds to α_1_ < α < α_2_, and the third column corresponds to α > α_2_, where α_1_ and α_2_ are the solutions for E[*Z*_*n*_*Z*_*n*−*k*_] = 0 for a given parameter γ.

### 3.2. Influence of α and γ on the coincidence count distribution

**Figure 8:**
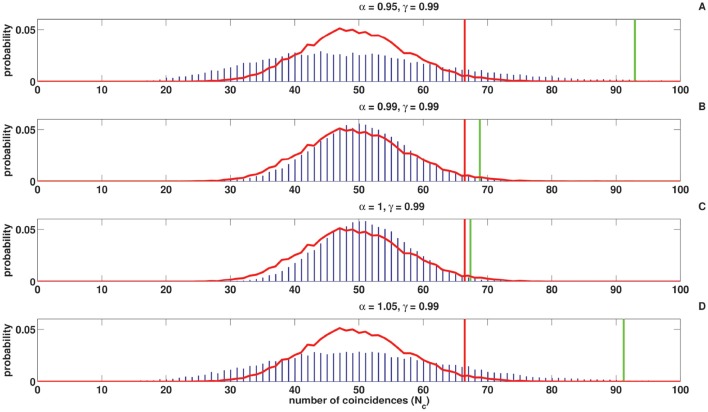
**Coincidence count distribution for the spike trains generated from the C-log-normal process with the parameters γ = 0.99 and α = [0.95, 0.99, 1, 1.05], indicated by the blue histogram**. The red profile indicates coincidence count distribution of the Poisson process. The red and green vertical lines show the critical numbers of coincidences that correspond to 1% significance level under the assumption that the underlying spike trains are generated from the Poisson and C-log-normal processes, respectively (*CV* = 1, *R* = 50*Hz*). **(A–D)** correspond to γ = 0.99 and different α.

**Figure 9:**
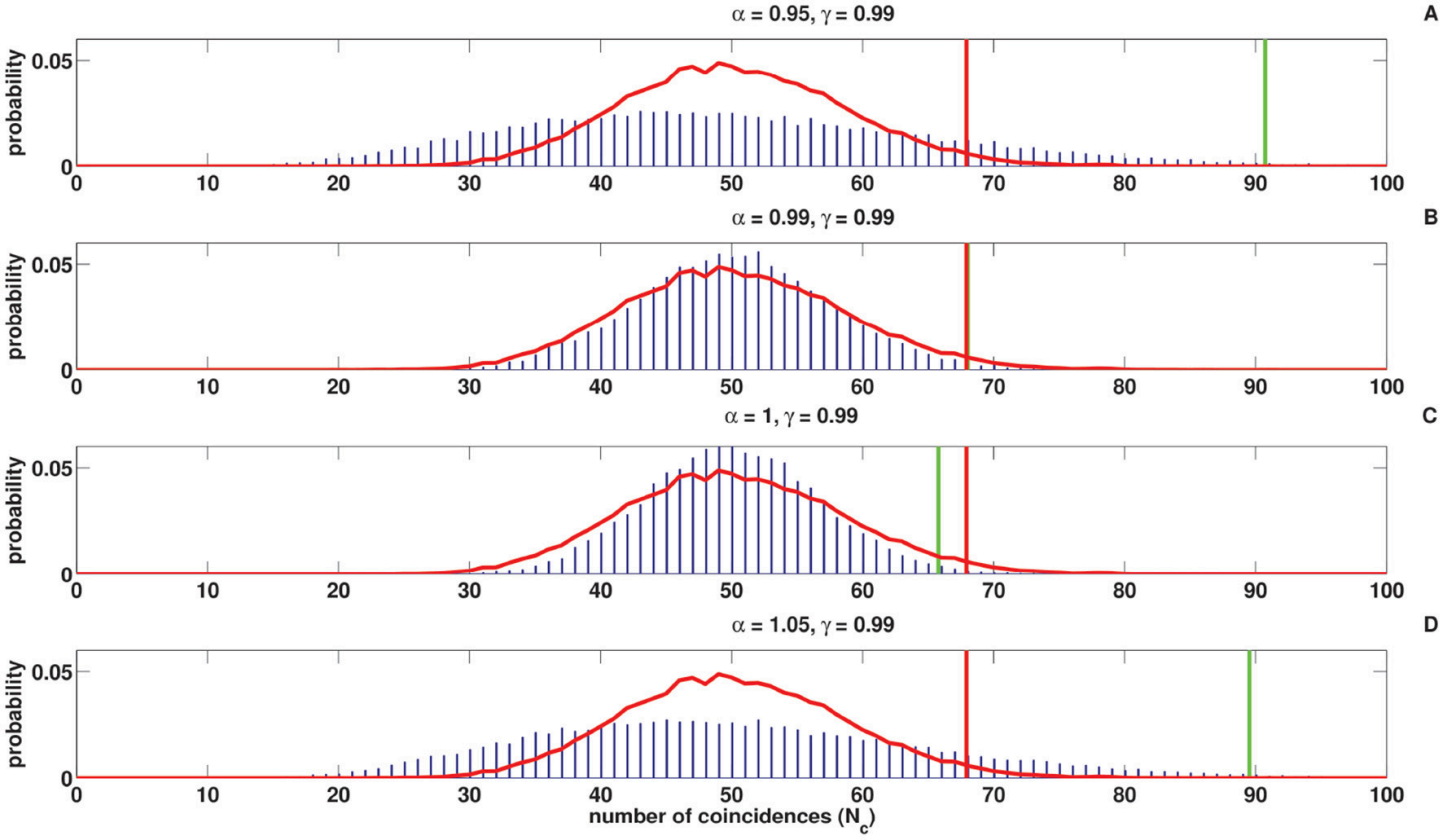
**Coincidence count distribution for the spike trains generated from the C-log-normal process with the parameters γ = 0.99 and α = [0.95, 0.99, 1, 1.05], indicated by the blue histogram**. The red profile indicates the coincidence count distribution of the log-normal process. The red and green vertical lines show the critical numbers of coincidences that correspond to1% significance level under the assumption that the underlying spike trains are generated from the the log-normal and C-log-normal processes, respectively (*CV* = 1, *R* = 50*Hz*). **(A–D)** correspond to γ = 0.99 and different α.

Figures 8, 9 show the coincidence count distribution for the spike trains generated from the C-log-normal process with parameters γ = 0.99 and α = [0.95, 0.99, 1, 1.05]. The red profile in Figure 8 indicates the coincidence count distribution of the Poisson process and in Figure 9 indicates the coincidence count distribution of the log-normal process with the same firing rate as is chosen for the C-log-normal process, i.e., *R* = 50*Hz*. The green vertical line in both figures shows the critical number of coincidences that corresponds to 1% significance level under the assumption that the underlying spike trains are generated from the C-log-normal process and the red vertical line in Figures 8, 9 indicates the critical number of coincidences that corresponds to 1% significance level under the assumption that the underlying spike trains are generated from the Poisson process and the log-normal process, respectively. In Figures 8, 9A,D) (with α = 0.95 and α = 1.05 which are out of the interval [0.99, 1.01], two solutions of E[*Z*_*n*_*Z*_*n*−*k*_] = 0) the coincidence count distributions have long tail which results in a higher false positive rate. On the contrary, in Figure 9C (with α = 1 which is in the interval [0.99, 1.01]) the coincidence count distribution has a shorter tail and the false positive value is smaller. Figure 8B (with α = γ) also shows a shorter tail and a smaller false positive value, but in Figure 9B the red and green lines show the same critical number since the condition of α = γ implies that the C-log-normal process can be considered as the log-normal process.

### 3.3. Impact of α and γ on the false positive rate

**Figure 10:**
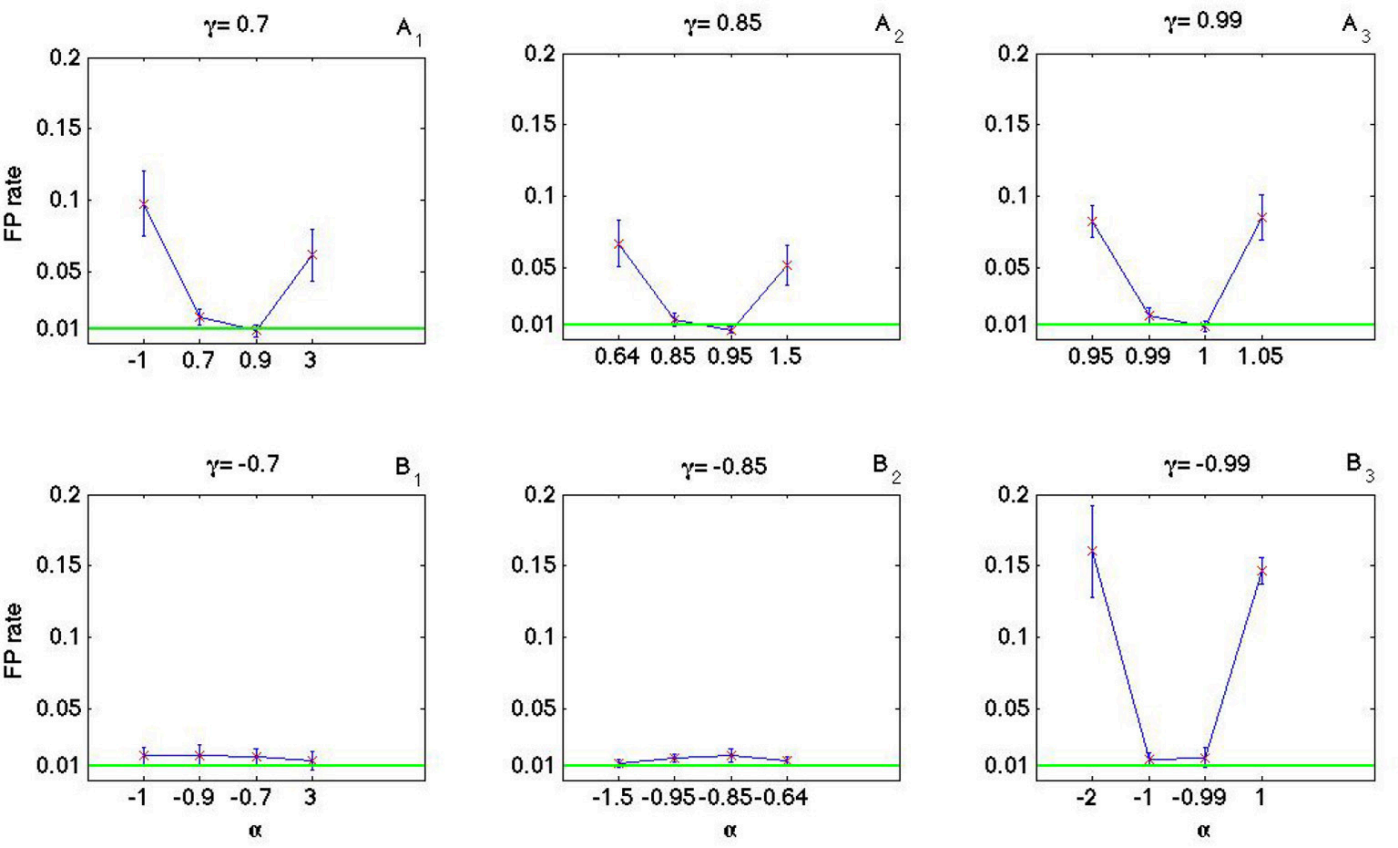
**False positive rate of the coincidence count distribution of the C-log-normal process based on the critical number of coincidences that corresponds to 1% significance level under the assumption that the underlying spike trains are from the Poisson process. (A_1_–A_3_)** correspond to positive γs and **(B**_1_**–B**_3_**)** correspond to negative γs (*CV* = 1, *R* = 50*Hz*).

**Figure 11:**
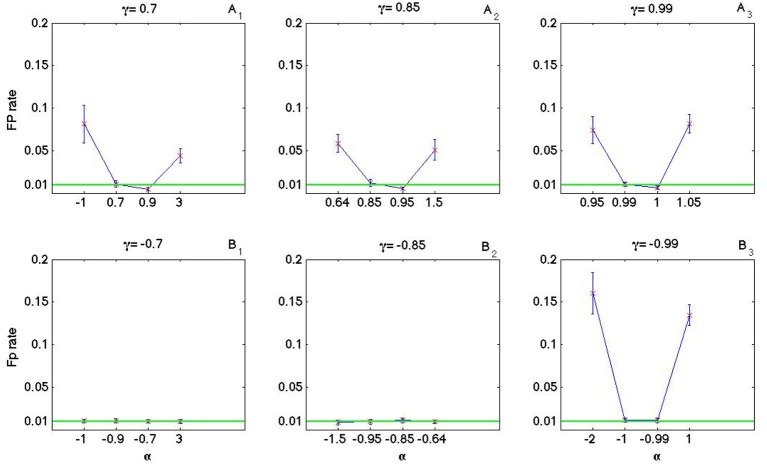
**False positive rate of the coincidence count distribution of the C-log-normal process based on the critical number of coincidences that corresponds to 1% significance level under the assumption that the underlying spike trains are from the log-normal process. (A_1_–A_3_)** correspond to positive γs and **(B**_1_**–B**_3_**)** correspond to negative γs (*CV* = 1, *R* = 50*Hz*).

As it is shown in Figures 8, 9 the coincidence count distribution for αs out of the interval [α_1_, α_2_] are more heavily tailed and for αs in the interval [α_1_, α_2_] the tail of coincidence count distribution of C-log-normal is shorter than the tail of coincidence count distribution of the Poisson and the log-normal processes. The heavy tailed coincidence count distribution results in strong consequences for hypothesis testing. Figures 10, 11 quantitatively compare the false positive rate of the coincidence count distribution of the C-log-normal with the Poisson process and the log-normal process, respectively. In Figure 10, the critical number of coincidences that corresponds to 1% significance level under the assumption that the underlying spike trains are Poissonian, is first estimated and based on this number, the false positive rate of the coincidence count distribution of the C-log-normal process is computed. In Figure 11, instead of the Poisson process, the false positive rates of the coincidence count distribution of the C-log-normal process are computed, based on the critical number of coincidences that corresponds to 1% significance level under the assumption that the underlying spike trains are from the log-normal process. In both Figures 10, 11, when α is out of the interval [α_1_, α_2_], then the false positive rates are more than 1% (for both γ > 0 and γ < 0), except in Figure 11B_1_ when γ = −0.7. When α is in the interval [α_1_, α_2_] and γ > 0, then the false positive is decreasing. However, if γ < 0 the false positive rate is again increasing. Moreover, in Figure 11, when α = γ, the false positive rate is close to 1%, i.e., it equals the false positive of the coincidence count distribution under the assumption that the spike trains are generated from the log-normal process.

### 3.4. Comparison of the full coincidence count distribution

**Figure 12:**
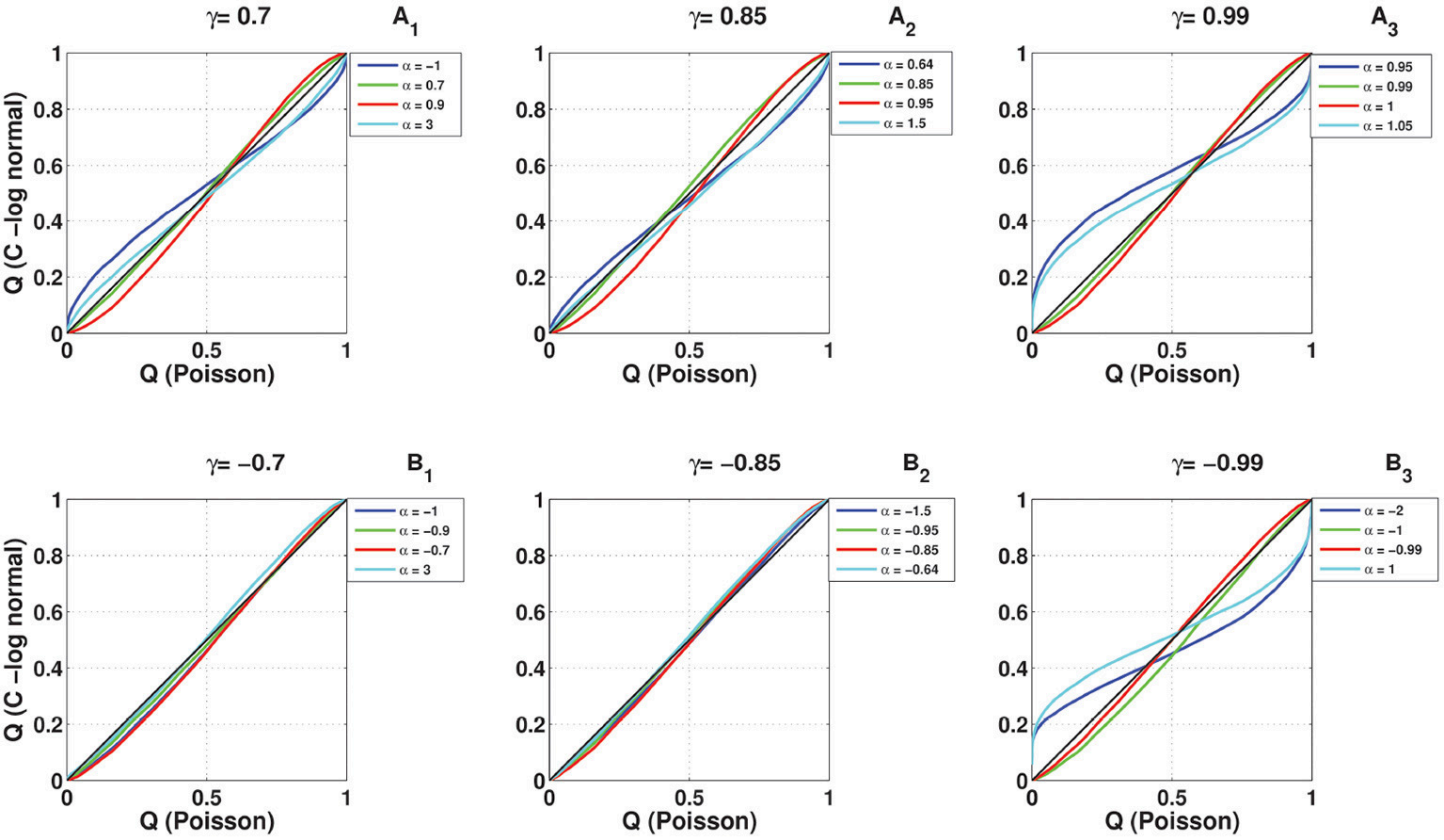
**Quantile-Quantile (QQ) plots of the coincidence count distribution of the Poisson and C-log-normal processes (*CV* = 1, *R* = 50*Hz*)**. **(A**_1_**–A**_3_**)** correspond to γ > 0 and different α. **(B**_1_**–B**_3_**)** correspond to γ < 0 and different α.

**Figure 13:**
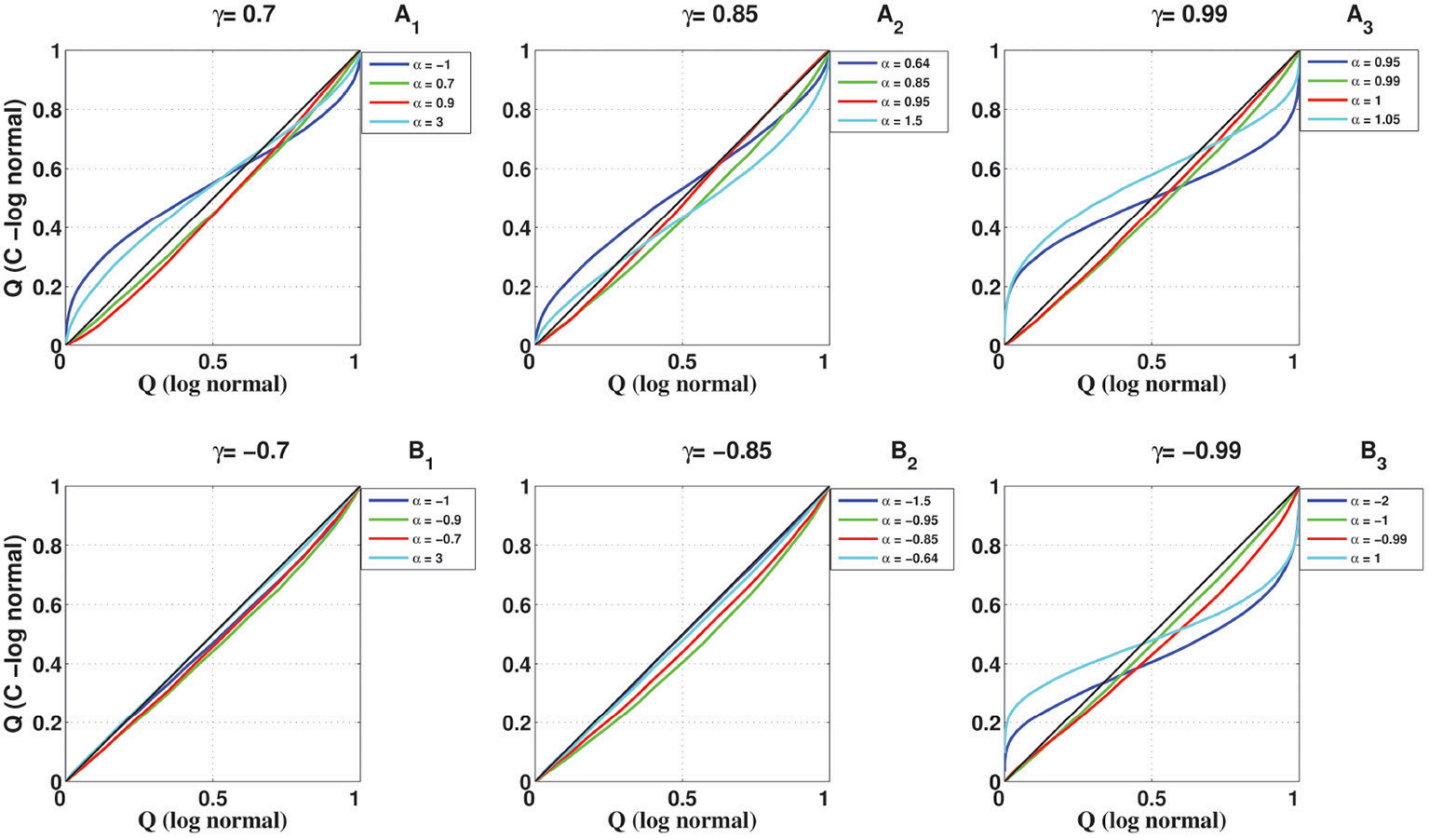
**Quantile-Quantile (QQ) plots of the coincidence count distribution of the log-normal and C-log-normal processes (*CV* = 1, *R* = 50*Hz*)**. **(A**_1_**–A**_3_**)** correspond to γ > 0 and different α. **(B**_1_**–B**_3_**)** correspond to γ < 0 and different α.

To compare the full distribution of the coincidence count of the C-log-normal process with the coincidence count distribution of the Poisson process and the log-normal process, quantile-quantile (QQ) plots are shown in Figures 12, 13, respectively. The qualitative behavior of plots in both Figures 12, 13 are the same. The higher the magnitude of γ, the more the curve diverges from the diagonal. Also, in each panel where the magnitude of γ is constant, the value of α affects how much the curve diverges from the diagonal. The further α is away from one side of the interval [α_1_, α_2_], the more the curve diverges from the diagonal. For example, in Figures 12A_3_, 13A_3_, α = 0.95, 1.05 are both out of the interval [α_1_ = 0.99, α_2_ = 1.01] and the divergence of their corresponding curves is strongly pronounced. For α = 1, which is in the interval [0.99, 1.01], the divergence is not very pronounced since its distance from α_1_ or α_2_ is not high. The curve corresponding to α = 0.99 lies on the diagonal because when α = γ the C-log-normal process can be considered as the log-normal process, thus both have the same coincidence count distribution. In both figures, on the bottom left of the panels, any curve above the diagonal indicates an increased false-positive level, and below the diagonal indicates a reduced number of false positives if the test statistics is based on the assumption that spike trains follow Poissonian firing or log-normal ISIs. In contrast, on the top right of the panels, any curve below the diagonal indicates an increased false-positive level and above the curve indicates a decreased false positive rate.

## 4. Discussion

The discussion of whether the coordinated neural activities really exist and occur more often than expected by chance, in other words, whether they occur more often than is expected if the neurons fire independently has long tantalized neuroscientists.

To examine this issue, different approaches have been taken, which, most of them intentionally destroying or involuntarily neglecting the autostructure of the spiking activity of individual neurons. To analyze the simultaneous spike trains for precise spike correlation and test whether the observed coincidence events occur significantly above chance, many of these approaches model the spike train as a Poisson Process, implying that the generation of each spike is independent of all the other spikes, and the inter-spike intervals (ISIs) has an exponential distribution (Grün et al., 2002a,b). However, the experimental ISIs show substantial deviation from these assumptions. For example, they exhibit dependence among spike sequences, such as absolute and relative refractory periods, or bursting, periodic, or regular behaviors. Additionally, spike times might show higher order dependence of spike times lying further in the past, and thus cannot be modeled by the Poisson process.

In this paper, we have studied the influence of higher order dependence of spike times which lie further in the past and exist in the autostructure of the spike times, on the shape of the coincidence count distribution of pairs of mutually independent spike trains. To this end, we proposed a non-renewal process (denoted C-log-normal process) which is a generalized model of a renewal log-normal process. We derived the properties of C-log-normal process analytically. In addition, we used the Monte Carlo estimation to examine the effects of the model's parameters, α and γ, on the shape of the coincidence count distribution of pairs of mutually independent spike trains generated from the C-log-normal process. The results were also compared with the shape of the coincidence count distribution of pairs of mutually independent spike trains generated from the log-normal process and the Poisson process.

The first finding is that the sign of γ causes E[*Z*_*n*_*Z*_*n*−*k*_] to be either positive or negative (if γ > 0), or oscillates between positive and negative values (if γ < 0). If γ > 0 and α_1_ < α < α_2_, then E[*Z*_*n*_*Z*_*n*−*k*_] is negative, which results in the long inter-spike intervals to be followed by the short inter-spike intervals and vice versa. If α < α_1_ or α_2_ < α, then E[*Z*_*n*_*Z*_*n*−*k*_] is positive, that is, long inter-spike intervals are intended to be followed by long inter-spike intervals and vice versa. However, if γ < 0, then E[*Z*_*n*_*Z*_*n*−*k*_] shows oscillatory behavior, that is, if α_1_ < α < α_2_, then E[*Z*_*n*_*Z*_*n*−*k*_] oscillates from a positive to a negative value and if α < α_1_ or α_2_ < α, then E[*Z*_*n*_*Z*_*n*−*k*_] oscillates from a negative to a positive value.

The second finding is that, compared to the coincidence distributions of homogeneous Poisson processes and also non-Poisson processes, the width of the distribution of joint spike events of the C-log-normal process changes. The non-renewal C-log-normal process can lead to both heavy tailed or narrow coincidence distribution, which results in higher or lower false positive rates, respectively, in relation to 1% significance level under the assumption that the underlying spike trains from the Poisson or the log-normal process. If γ > 0 and α_1_ < α < α_2_ the false positive rate is decreased and if α < α_1_ or α_2_ < α the false positive rate is increased. The impact of γ < 0 is more complex and does not exactly follow the same behavior for different values of γ. However, for a higher magnitude of γ (e.g., γ = −0.99), the false positive rate when α_1_ < α < α_2_ is decreased and if α < α_1_ or α_2_ < α the false positive rate is increased.

In this study, the other parameters that can affect the autostructure of spike trains, namely the coefficient of variation of the inter-spike interval distributions *C*_*V*_ and the Fano factor *FF* were kept constant. These two parameters also have an impact on the probability distribution of joint spikes events (Pipa et al., 2013). In future work, the effects of these two factors along with the parameters of the C-log-normal process on the autostructure of spike trains, coincidence count distribution and false positive rate can be studied.

Additionally, the complexity of interactions between neurons can be extended. So far, we have discussed the effects of the autostructure of spike trains on the coincidence count distribution across pairs of neurons. In future work, this can be extended to higher complexities, such as triplet, quintuplet, or in general ζ-tuplet coincidences.

Another future direction worth pursuing is to use the C-log-normal process for modeling the experimental data. To this end, a method to fit the parameters of C-log-normal process, namely α and γ, needs to be developed.

In conclusion, the simulations done in this paper highlight the possible issues when spike trains deviate from Poisson but Poisson is assumed. In respect to the neural code, the lesson to learn is: do not make such a strong assumption about the data since it can make the analysis fragile. That is, the higher order dependence of spike times which lie further in the past can affect the autostructure of spike times, which can falsify the statistical inference of the existence of coordinated neuronal activity. This effect results in over or underestimation of statistical significancies.

## Author contributions

MS performed the calculus, simulations and wrote the original draft. CV provided the initial spike train model. GP was involved in the simulation, calculus and writing.

## Funding

We acknowledge support by Deutsche Forschungsgemeinschaft (DFG) and Open Access Publishing Fund of Osnabrück University.

### Conflict of interest statement

The authors declare that the research was conducted in the absence of any commercial or financial relationships that could be construed as a potential conflict of interest.

## Footnotes

^1^α_1_ and α_2_ are two solutions for equation E[*Z*_*n*_*Z*_*n*−*k*_] = 0
