# Supplementary material for: Serial Spike Time Correlations Affect Probability Distribution of Joint Spike Events
Source: Front Comput Neurosci. 2016 Dec 23;10:139. doi: 10.3389/fncom.2016.00139 (PMC5180579; doi:10.3389/fncom.2016.00139)
Supplement: Supplementary file 1 [file Presentation1.pdf]

Supplementary Material: Serial Spike Time Correlations Affect Probability Distribution of Joint Spike Events

Mina Shahi, Carl van Vreeswijk and Gordon Pipa*

*Correspondence:
Gordon Pipa:
gpipa@uos.de

1 FIGURES

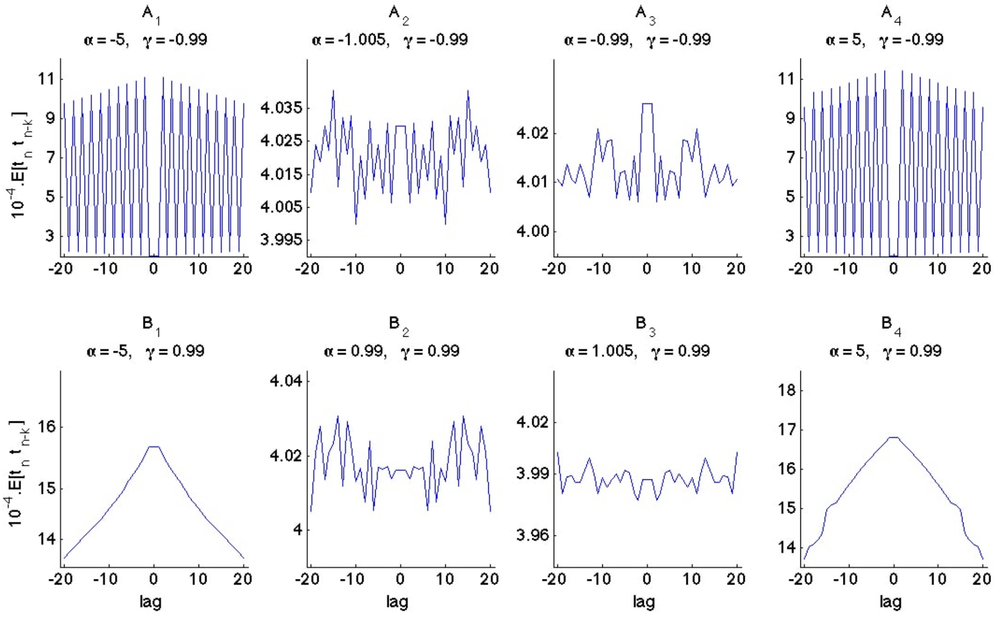

Figure S1. $\mathrm{E}[t_n t_{n-k}]$, the autocorrelation of the spike times. 200 mutually independent spike trains are generated from C-log-normal process for the same pairs of γ and α used in Figure 4 ($CV = 1$, $R = 50\,Hz$). The behaviors of the autocorrelation of $\mathrm{E}[t_n t_{n-k}]$ and $\mathrm{E}[Z_n Z_{n-k}]$ are qualitatively similar.

2 DERIVATIONS

2.1 Claim 1

Claim 1: If $X_{n-1} \sim N(0,1)$ and $\zeta_n \sim N\left(0, 1-\gamma^2\right)$ are two independent random variables, then $X_n \sim N(0,1)$ where:

$$X_n = \gamma X_{n-1} + \zeta_n \qquad |\gamma| < 1 \tag{S1}$$

Proof: If $X \sim N(\mu_X, \sigma_X^2)$ and $Y \sim N(\mu_Y, \sigma_Y^2)$ are independent random variables drawn from a normal distribution, then $Z = aX + bY$ is a normally distributed random variable with $\mu_Z = a\mu_X + b\mu_Y$ and $\sigma_Z^2 = a^2\sigma_X^2 + b^2\sigma_Y^2$, i.e., $Z \sim N(\mu_Z, \sigma_Z^2)$. Since X_{n-1} and ζ_n are two independent random variables drawn from a normal distribution, then $X_n = \gamma X_{n-1} + \zeta_n$ is also a normally distributed random variable

and its mean and variance are as follows:

$$\begin{aligned} \mathrm{E}[X_n] &= \mathrm{E}\left[\gamma X_{n-1} + \zeta_n\right] \\ &= \mathrm{E}[\gamma X_{n-1}] + \mathrm{E}[\zeta_n] \\ &= \gamma\, \mathrm{E}[X_{n-1}] + \mathrm{E}[\zeta_n] \\ &= 0; \end{aligned} \tag{S2}$$

since $\mathrm{E}[X_{n-1}] = 0$, and $\mathrm{E}[\zeta_n] = 0$.

$$\begin{aligned} \mathrm{Var}[X_n] &= \mathrm{Var}\left[\gamma X_{n-1} + \zeta_n\right] \\ &= \mathrm{Var}[\gamma X_{n-1}] + \mathrm{Var}[\zeta_n] \\ &= \gamma^2\ \mathrm{Var}[X_{n-1}] + \mathrm{Var}[\zeta_n] \\ &= \gamma^2 + \left(1 - \gamma^2\right) \\ &= 1; \end{aligned} \tag{S3}$$

since $\mathrm{Var}[X_{n-1}] = 1$, and $\mathrm{Var}[\zeta_n] = 1 - \gamma^2$.

2.2 Claim 2

Claim 2: If $X_n \sim N(0,1)$, $X_{n-1} \sim N(0,1)$ and $\zeta_n \sim N\left(0, 1-\gamma^2\right)$ and $X_n = \gamma\, X_{n-1} + \zeta_n$, $|\gamma| < 1$, then $Z_n \sim N(0,1)$ where:

$$Z_n = \frac{X_n - \alpha\, X_{n-1}}{\sqrt{1 + \alpha^2 - 2\alpha\gamma}}, \qquad (\alpha(\alpha - 2\gamma) > -1) \tag{S4}$$

Proof:

$$\begin{aligned} Z_n &= \frac{X_n - \alpha\, X_{n-1}}{\sqrt{1 + \alpha^2 - 2\alpha\gamma}} \\ &= \frac{\gamma\, X_{n-1} + \zeta_n - \alpha\, X_{n-1}}{\sqrt{1 + \alpha^2 - 2\alpha\gamma}} \\ &= \frac{(\gamma - \alpha) X_{n-1} + \zeta_n}{\sqrt{1 + \alpha^2 - 2\alpha\gamma}}; \end{aligned} \tag{S5}$$

and since X_{n-1} and ζ_n are two independent, normally distributed random variables, Z_n which is a linear combination of these two variables is also a normally distributed ranodm variable. The derivations below

give the mean and variance of Z_n:

$$\begin{aligned}
\mathrm{E}[Z_n] &= \mathrm{E}\left[\frac{X_n - \alpha X_{n-1}}{\sqrt{1+\alpha^2-2\alpha\gamma}}\right] \\
&= \frac{1}{\sqrt{1+\alpha^2-2\alpha\gamma}}\,\mathrm{E}[X_n - \alpha\, X_{n-1}] \\
&= \frac{1}{\sqrt{1+\alpha^2-2\alpha\gamma}}\,\mathrm{E}\left[\gamma\, X_{n-1} + \zeta_n - \alpha\, X_{n-1}\right] \\
&= \frac{1}{\sqrt{1+\alpha^2-2\alpha\gamma}}\left((\gamma-\alpha)\ \mathrm{E}[X_{n-1}] + \mathrm{E}[\zeta_n]\right) \\
&= 0;
\end{aligned} \tag{S6}$$

since $\mathrm{E}[X_n] = \mathrm{E}[\zeta_n] = 0$.

$$\begin{aligned}
\mathrm{Var}[Z_n] &= \mathrm{Var}\left[\frac{X_n - \alpha\, X_{n-1}}{\sqrt{1+\alpha^2-2\alpha\gamma}}\right] \\
&= \frac{1}{1+\alpha^2-2\alpha\gamma}\,\mathrm{Var}[X_n - \alpha\, X_{n-1}] \\
&= \frac{1}{1+\alpha^2-2\alpha\gamma}\,\mathrm{Var}\left[\gamma\, X_{n-1} + \zeta_n - \alpha\, X_{n-1}\right] \\
&= \frac{1}{1+\alpha^2-2\alpha\gamma}\left((\gamma-\alpha)^2\ \mathrm{Var}[X_{n-1}] + \mathrm{Var}[\zeta_n]\right) \\
&= \frac{1}{1+\alpha^2-2\alpha\gamma}\left((\gamma-\alpha)^2 + \left(1-\gamma^2\right)\right) \\
&= 1;
\end{aligned} \tag{S7}$$

since $\mathrm{Var}[X_n] = 1$ and $\mathrm{Var}[\zeta_n] = 1 - \gamma^2$.

2.3 Claim 3

Claim 3: If X_n, X_{n-1}, ζ_n and Z_n are random variables defined according to *Claim 2* then:

$$\langle Z_n\, Z_{n-k}\rangle = \gamma^{|k|-1}\left(\frac{(1+\alpha^2)\,\gamma - \alpha\,(1+\gamma^2)}{1+\alpha^2-2\alpha\gamma}\right), \qquad k \neq 0 \tag{S8}$$

Proof:

$$
\begin{aligned}
\langle Z_n\, Z_{n-k}\rangle &= \mathrm{E}\left[\left(\frac{X_n-\alpha\,X_{n-1}}{\sqrt{1+\alpha^2-2\alpha\gamma}}\right)\left(\frac{X_{n-k}-\alpha\,X_{n-k-1}}{\sqrt{1+\alpha^2-2\alpha\gamma}}\right)\right]\\
&= \mathrm{E}\left[\frac{X_nX_{n-k}-\alpha X_nX_{n-k-1}-\alpha X_{n-1}X_{n-k}+\alpha^2X_{n-1}X_{n-k-1}}{1+\alpha^2-2\alpha\gamma}\right]\\
&= \left[\frac{\mathrm{E}\left[X_n\,X_{n-k}\right]-\alpha\,\mathrm{E}\left[X_n\,X_{n-k-1}\right]-\alpha\,\mathrm{E}\left[X_{n-1}\,X_{n-k}\right]+\alpha^2\,\mathrm{E}\left[X_{n-1}\,X_{n-k-1}\right]}{1+\alpha^2-2\alpha\gamma}\right]\\
&= \frac{\gamma^{\,|k|}-\alpha\gamma^{\,|k|+1}-\alpha\gamma^{\,|k|-1}+\alpha^2\gamma^{\,|k|}}{1+\alpha^2-2\alpha\gamma}\\
&= \gamma^{\,|k|-1}\left(\frac{(1+\alpha^2)\,\gamma-\alpha\,(1+\gamma^2)}{1+\alpha^2-2\alpha\gamma}\right)\\
&= \gamma^{\,|k|-1}\,C(\alpha,\gamma).
\end{aligned}
\tag{S9}
$$

To obtain the last three equations we need to derive the values of $\mathrm{E}\left[X_n\,X_{n-k}\right]$, $\mathrm{E}\left[X_n\,X_{n-k-1}\right]$, $\mathrm{E}\left[X_{n-1}\,X_{n-k}\right]$ and $\mathrm{E}\left[X_{n-1}\,X_{n-k-1}\right]$ given in equation S9. First X_n is expanded in terms of X_{n-k}, ζ_{n-k}, $\zeta_{n-(|k|+1)}$,. . ., ζ_{n-1} and ζ_n.

Claim I: Let $X_n = \gamma\,X_{n-1}+\zeta_n$ then X_n can be expanded as follows:

$$
X_n = \gamma^{|k|}\,X_{n-|k|}+\gamma^{|k|-1}\zeta_{n-(|k|-1)}+\cdots+\zeta_n \tag{S10}
$$

Proof: We prove the claim by induction. As it is given in the assumption of the claim for $k=1$,

$$
X_n = \gamma\,X_{n-1}+\zeta_n
$$

let $k=s, s>0$ then

$$
X_n = \gamma^{|s|}\,X_{n-|s|}+\gamma^{|s|-1}\zeta_{n-(|s|-1)}+\cdots+\zeta_n
$$

we show that for $k=s+1$, (if $s<0$ then $k=s-1$), the following holds

$$
X_n = \gamma^{|s|+1}X_{n-(|s|+1)}+\gamma^{|s|}\zeta_{n-|s|}+\gamma^{|s|-1}\zeta_{n-(|s|-1)}+\cdots+\zeta_n
$$

$$
\begin{aligned}
X_n &= \gamma^{|s|}\,X_{n-|s|}+\gamma^{|s|-1}\zeta_{n-(|s|-1)}+\cdots+\zeta_n\\
&= \gamma^{|s|}\left(\gamma X_{n-(|s|+1)}+\zeta_{n-|s|}\right)+\gamma^{|s|-1}\zeta_{n-(|s|-1)}+\cdots+\zeta_n\\
&= \gamma^{|s|+1}X_{n-(|s|+1)}+\gamma^{|s|}\zeta_{n-|s|}+\gamma^{|s|-1}\zeta_{n-(|s|-1)}+\cdots+\zeta_n
\end{aligned}
\tag{S11}
$$

For example, X_n can be expanded in terms of X_{n-3}, ζ_{n-2}, ζ_{n-1} and ζ_n as follows:

$$
\begin{aligned}
X_n &= \gamma\,X_{n-1}+\zeta_n\\
&= \gamma\left(\gamma X_{n-2}+\zeta_{n-1}\right)+\zeta_n\\
&= \gamma\left(\gamma\left(\gamma\,X_{n-3}+\zeta_{n-2}\right)+\zeta_{n-1}\right)+\zeta_n\\
&= \gamma^3X_{n-3}+\gamma^2\zeta_{n-2}+\gamma\zeta_{n-1}+\zeta_n
\end{aligned}
\tag{S12}
$$

Now we use *Claim I* to find the value of $\mathrm{E}\left[X_n\, X_{n-s}\right]$ as follows:

$$\begin{aligned}
\mathrm{E}\left[X_n\, X_{n-s}\right] &= \mathrm{E}\left[\left(\gamma^{|s|}\, X_{n-|s|} + \gamma^{|s|-1}\zeta_{n-(|s|-1)} + \cdots + \zeta_n\right) X_{n-s}\right] \\
&= \mathrm{E}\left[\gamma^{|s|} X_{n-s}^2 + \gamma^{|s|-1}\zeta_{n-(|s|-1)}\, X_{n-s} + \cdots + \zeta_n\, X_{n-s}\right] \\
&= \gamma^{|s|}\ \mathrm{E}\left[X_{n-s}^2\right] + \gamma^{|s|-1}\,\mathrm{E}\left[X_{n-s}\,\zeta_{n-(|s|-1)}\right] + \cdots + \\
&\mathrm{E}\left[X_{n-s}\,\zeta_n\right] \\
&= \gamma^{|s|}
\end{aligned} \tag{S13}$$

To obtain the last equation we need to know the values of $\mathrm{E}\left[X_{n-s}^2\right]$, $\mathrm{E}\left[X_{n-s}\,\zeta_{n-(|s|-1)}\right]$,. . ., $\mathrm{E}\left[X_{n-s}\,\zeta_n\right]$ which are given as follows:

$$\begin{aligned}
\mathrm{E}\left[X_{n-s}^2\right] &= \mathrm{Var}\left[X_{n-s}\right] + \left(\mathrm{E}\left[X_{n-s}\right]\right)^2 \\
&= 1;
\end{aligned} \tag{S14}$$

since according to *Claim 1*, $\mathrm{E}\left[X_{n-s}\right] = 0$ and $\mathrm{Var}\left[X_{n-s}\right] = 1$.

$$\mathrm{E}\left[X_{n-s}\,\zeta_n\right] = \mathrm{E}\left[X_{n-s}\right]\ \mathrm{E}\left[\zeta_n\right] = 0; \tag{S15}$$

since according to *Claim 1* X_{n-s} and ζ_n are independent variables and $\mathrm{E}\left[\zeta_n\right] = \mathrm{E}\left[X_{n-s}\right] = 0$.
Also with the same argument

$$\mathrm{E}\left[X_{n-s}\,\zeta_{n-(|s|-1)}\right] = \mathrm{E}\left[X_{n-s}\right]\ \mathrm{E}\left[\zeta_{n-(|s|-1)}\right] = 0 \tag{S16}$$

By substituting these values the last equation of S13 is obtained. We can now apply the result of equation S13 to find the value of $\mathrm{E}\left[X_{n-k}\, X_{n-s}\right]$ for arbitrary s and k. To obtain equation S9 we need the values of $\mathrm{E}\left[X_n\, X_{n-k}\right]$, $\mathrm{E}\left[X_n\, X_{n-k-1}\right]$, $\mathrm{E}\left[X_{n-1}\, X_{n-k}\right]$ and $\mathrm{E}\left[X_{n-1}\, X_{n-k-1}\right]$, which are as follows:

$$\mathrm{E}\left[X_n\, X_{n-k}\right] = \gamma^{\,|k|} \tag{S17}$$

$$\mathrm{E}\left[X_n\, X_{n-k-1}\right] = \begin{cases} \gamma^{\,|k|+1}, & \text{if } k \geq 0 \\ \gamma^{\,|k|-1}, & \text{if } k < 0 \end{cases} \tag{S18}$$

$$\mathrm{E}\left[X_{n-1}\, X_{n-k}\right] = \begin{cases} \gamma^{\,|k|-1}, & \text{if } k \geq 0 \\ \gamma^{\,|k|+1}, & \text{if } k < 0 \end{cases} \tag{S19}$$

$$\mathrm{E}\left[X_{n-1}\, X_{n-k-1}\right] = \gamma^{\,|k|} \tag{S20}$$
