# Supplementary figures and images for: Serial Spike Time Correlations Affect Probability Distribution of Joint Spike Events
Source: Front Comput Neurosci. 2016 Dec 23;10:139. doi: 10.3389/fncom.2016.00139 (PMC5180579; doi:10.3389/fncom.2016.00139)

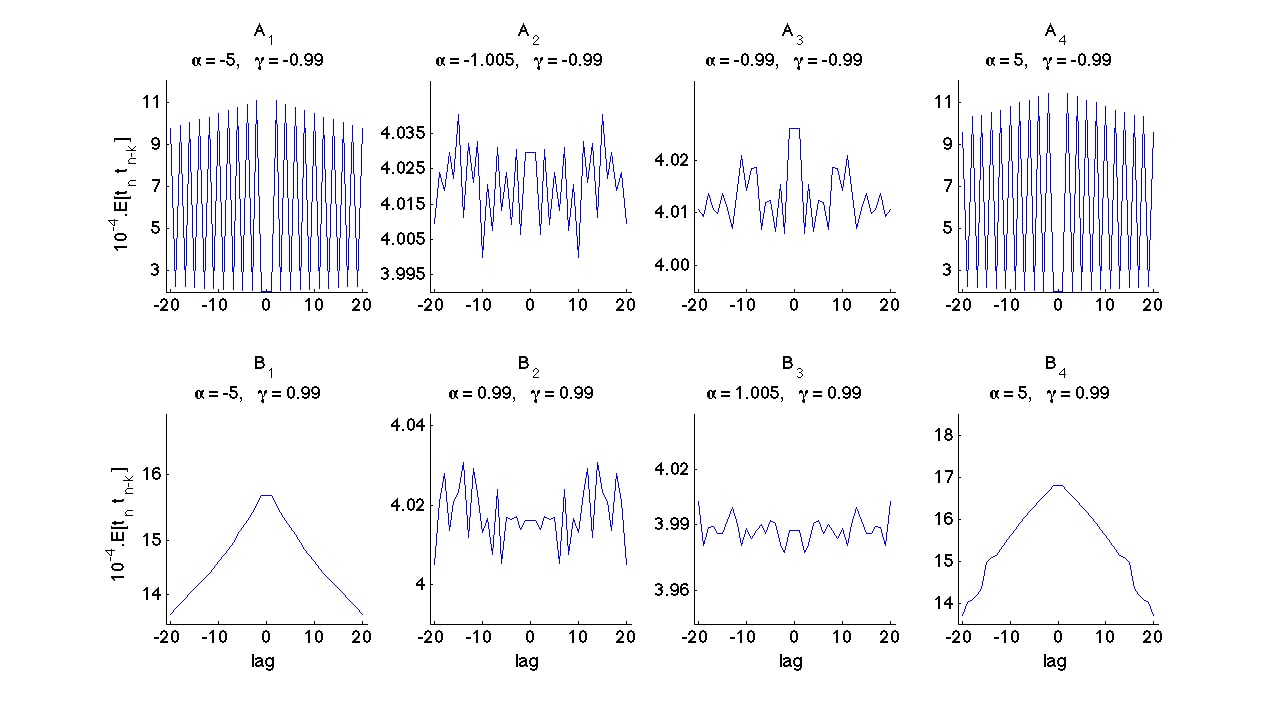

Supplement: Supplementary file 2 [file Image1.JPEG]
